# Cardiovascular Magnetic Resonance Imaging Patterns in Rare Cardiovascular Diseases

**DOI:** 10.3390/jcm11216403

**Published:** 2022-10-29

**Authors:** George Markousis-Mavrogenis, Aikaterini Giannakopoulou, Antonios Belegrinos, Maria Roser Pons, Maria Bonou, Vasiliki Vartela, Antigoni Papavasiliou, Aikaterini Christidi, Soultana Kourtidou, Genovefa Kolovou, Flora Bacopoulou, George P. Chrousos, Sophie I. Mavrogeni

**Affiliations:** 1 Onassis Cardiac Surgery Center, 17674 Athens, Greece; 2 Aghia Sophia Children’s Hospital, 11527 Athens, Greece; 3 Laikon Hospital, 11527 Athens, Greece; 4 Iaso Children’s Hospital, 15123 Athens, Greece; 5 Euromedica MRI Unit, 54645 Thessaloniki, Greece; 6 University Research Institute for Maternal and Child Health and Precision Medicine, National and Kapodistrian University of Athens, 11527 Athens, Greece; 7 Center for Adolescent Medicine and UNESCO Chair on Adolescent Health Care, First Department of Pediatrics, “Agia Sophia” Children’s Hospital, School of Medicine, National and Kapodistrian University of Athens, 11527 Athens, Greece

**Keywords:** cardiovascular, cardiovascular magnetic resonance, magnetic resonance imaging, cardiac, MRI, rare disease, heart, imaging, CMR, rare cardiovascular disease

## Abstract

Rare cardiovascular diseases (RCDs) have low incidence but major clinical impact. RCDs’ classification includes Class I—systemic circulation, Class II—pulmonary circulation, Class III—cardiomyopathies, Class IV—congenital cardiovascular diseases (CVD), Class V—cardiac tumors and CVD in malignancy, Class VI—cardiac arrhythmogenic disorders, Class VII—CVD in pregnancy, Class VIII—unclassified rare CVD. Cardiovascular Magnetic Resonance (CMR) is useful in the diagnosis/management of RCDs, as it performs angiography, function, perfusion, and tissue characterization in the same examination. Edema expressed as a high signal in STIRT2 or increased T2 mapping is common in acute/active inflammatory states. Diffuse subendocardial fibrosis, expressed as diffuse late gadolinium enhancement (LGE), is characteristic of microvascular disease as in systemic sclerosis, small vessel vasculitis, cardiac amyloidosis, and metabolic disorders. Replacement fibrosis, expressed as LGE, in the inferolateral wall of the left ventricle (LV) is typical of neuromuscular disorders. Patchy LGE with concurrent edema is typical of myocarditis, irrespective of the cause. Cardiac hypertrophy is characteristic in hypertrophic cardiomyopathy (HCM), cardiac amyloidosis (CA) and Anderson–Fabry Disease (AFD), but LGE is located in the IVS, subendocardium and lateral wall in HCM, CA and AFD, respectively. Native T1 mapping is increased in HCM and CA and reduced in AFD. Magnetic resonance angiography provides information on aortopathies, such as Marfan, Turner syndrome and Takayasu vasculitis. LGE in the right ventricle is the typical finding of ARVC, but it may involve LV, leading to the diagnosis of arrhythmogenic cardiomyopathy. Tissue changes in RCDs may be detected only through parametric imaging indices.

## 1. Introduction

The commonly accepted prevalence of rare diseases is less than 1 per 2000 in the general population [1,2]. Although rare cardiovascular diseases (RCDs) have a low incidence, they have a major effect on the life of the affected patients and their families. RCDs affect around 30 million people in Europe, leading to disability and major socio-economic consequences. These conditions are rare and, therefore, a high level of expertise is necessary for diagnosis, risk stratification, and management [1].

The proposed classification of RCDs by Podolec et al. is presented in Table 1 [3].

Taking into consideration that there is only limited access to specific cardiac investigations the diagnosis of RCDs remains challenging. In most cases, suspicion is raised by an unusual or extraordinary event—for example, a sudden death or disability in a young relative. In other cases, the diagnosis is retrospective, following delayed recognition of atypical signs. Although the diagnosis of RCDs requires some specialized knowledge and access to genetic testing, the clinical assessment combined with cardiovascular imaging can be used as an initial filter to detect some of the most important RCDs and to motivate further investigation.

The main cardiovascular imaging modalities for RCDs evaluation include echocardiography (echo) and cardiovascular magnetic resonance (CMR) and in some cases nuclear techniques such as SPECT and PET. Echocardiography represents the cornerstone of imaging in Cardiology. It is widely available, bedside, cost-effective, and without radiation, with great acceptance between the cardiologists. However, it is operator and acoustic window dependent modality, has limited field of view and cannot perform direct tissue characterization. SPECT and PET provide information regarding myocardial perfusion at rest and during stress, but they are expensive modalities, use ionizing radiation and have a lower spatial resolution, compared with echocardiography and CMR. More specifically, PET remains one of the most expensive modalities, is not widely available and needs a high level of expertise. Pet can also provide information regarding myocardial inflammation; however, the use of radiation limits its use for serial evaluation [4].

Our aim in this review is to present the role of CMR in the diagnosis/risk stratification of RCDs.

## 2. CMR Applications for Cardiovascular Diseases

**Figure 1:**
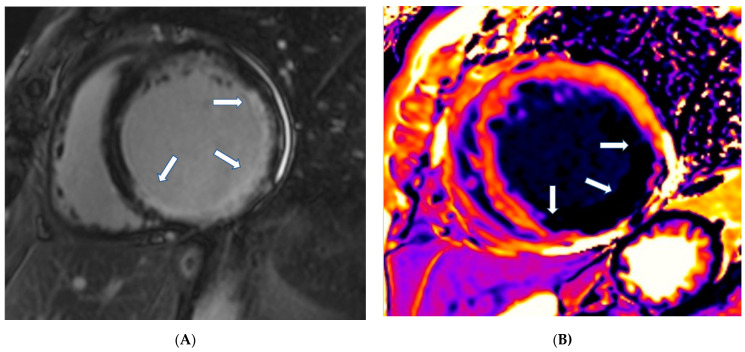
(**A**) Short-axis LGE image showing myocardial infarction (arrows) in the inferolateral wall of LV. (**B**) Matching native T1 mapping of the same patient.

CMR using various sequences can be of great value in the assessment of the pathophysiology in RCDs. More specifically, CMR can perform:

**1.**  **Assessment of cardiac function:** The CMR pulse sequence used for functional evaluation of the heart is the balanced steady-state free precession (bSSFP), which is the gold standard for the evaluation of cardiac anatomy, mass, wall motion, and atrial and ventricular function because it is a tomographic modality without clinical assumption of cardiac shape [5]. It is of great value specifically for the assessment of the right cardiac ventricle (RV), which is usually underestimated by echocardiography [5].
**2.**  **Assessment of pericardium**
  **(a)**  **Pericardial inflammation:** CMR using T1-W black blood and SSFP sequences allows for anatomical characterization of the pericardium. Native T1 and T2 mapping provide additional information regarding pericardial inflammation. The inflamed pericardium is enhanced after the use of paramagnetic contrast, being a key tool to assess pericardial inflammation [5]. Its persistence, despite standard medical treatment in symptomatic patients, supports the need for prolonged treatment [6].
  **(b)**  **Pericardial effusion:** CMR criteria for pericardial effusion characterization are based on the total amount of fluid in the pericardium. If the intrapericardial space anterior to RV on SSFP is <4 mm, ≥5 mm (100–500 mL) and >10–15 mm is considered as small, medium, or large, respectively. Native T1 mapping of the pericardial fluid provides information about its composition. A native T1 mapping cut-off value of 3013 ms can differentiate transudates from exudates with a sensitivity of 94% and specificity of 79%, [7] with lower values suggesting exudative pericardial effusions [7]. In addition, native T1 and T2 mapping may reveal coexisting myocardial inflammation/fibrosis. However, mapping techniques are sequence- and magnet-specific and therefore, unlike LGE, they remain more challenging for standardization depending on different magnetic fields and protocols used.
  **(c)**  **Constrictive Pericarditis:** In the appropriate clinical scenario, a thickened pericardium >4 mm, visualized with SSFP or LGE, is a potential indicator of constriction [8,9]. However, constriction is characterized by both anatomic and hemodynamic alterations and, therefore, the final diagnosis of constrictive pericarditis should be confirmed by cardiac catheterization. In this context, CMR can assess the characteristic S-shaped interventricular septum using SSFP, dilated inferior or superior vena cava and/or coronary sinus. Real-time cine may demonstrate the effect of free breathing on ventricular interdependence, which is the typical marker of constrictive pericarditis [9,10,11,12].
**3.**  **Myocardial tissue characterization.**
  **(a)**  **T1-weighted images (T1-W) and late gadolinium enhancement (LGE):** T1-W imaging provides information for a morphological assessment of the heart. Late gadolinium-enhanced T1-W images (LGE), taken 10–15 min after gadolinium-based contrast administrations using inversion recovery pulse sequences, allows for the detection of myocardial replacement fibrosis (scar) (Figure 1) [13]. LGE may also detect marked extracellular interstitial expansion in association with amyloidosis (amyloid deposition and fibrosis) and in pulmonary hypertension (myocardial disarray with increased collagen content without focal replacement fibrosis). In myocarditis, LGE mainly reflects inflammation, combined with or without fibrosis [13]. In the acute phase of myocarditis, LGE correlates with necrosis (associated with edema as assessed by T2 mapping), while in the chronic phase, it corresponds to fibrosis (with less or no edema) [13]. Thrombi (if not organized) do not accumulate contrast agents, making LGE ideal in excluding recent thrombi [14].
    Myocardial infarction is characterized by subendocardial or transmural LGE in the distribution of epicardial coronary arteries. Subepicardial or patchy LGE usually in the inferolateral wall is characteristic of myocarditis. Finally, diffuse subendocardial LGE that does not follow the typical distribution of epicardial coronary arteries is often associated with microvascular coronary artery disease, vasculitis, antiphospholipid syndrome, and endocrine disorders, such as Cushing syndrome and autoimmune thyroid disease [13].
  **(b)**  **T2-weighted images (T2-W):** T2-W images result from water accumulation, due to edema [15], reflecting an acute myocardial response to damage of either ischemic (myocardial infarction) or inflammatory (myocarditis) etiology. It may be localized or diffuse, subendocardial or transmural, following the distribution of epicardial coronary arteries, as in CAD, or subepicardial, as in myocarditis. It can also be diffuse subendocardial, as in microvascular coronary artery disease and vasculitis [13]. T2-W injury appears as a high signal intensity area on short tau inversion recovery (STIR T2) images, where the signal contrast between edema, normal myocardium, and LV cavity is optimum. The STIR T2 image limitations include poor contrast between healthy and edematous areas, due to low signal-to-noise ratio [13], dependency on magnetic field homogeneity, and slow flow hyperintensity with motion artifacts [13].
  **(c)**  **T2 mapping:** In T2 mapping a parametric image of each voxel is reconstructed to overcome the problems of STIR T2. At 1.5 T, normal myocardium T2 values have been reported as 52 ± 3 ms by Giri et al. [16] and 55 ± 5 by Wassmuth et al. [17]. T2 measures are independent of body surface area and/or heart rate and have good reproducibility; however, they may vary with different scanner types or field strengths [18]. Normal values are also dependent on topographical LV location with increasing values from base to apex [18]. Increased signal on T2 mapping indicates myocardial edema, due to a recent cardiac lesion, as opposed to fibrosis [13,16,17].
    We should note that T2-W evaluation is a qualitative approach, prone to many technical limitations, while T2 mapping is a precise quantitative approach providing information on each voxel.
  **(d)**  **Stress CMR:** Rapid cardiac imaging using T1-W after pharmacologic, hyperemic stress with adenosine (or dipyridamole, ATP, regadenosine) and bolus injection of paramagnetic Gd-based contrast agent provides accurate and reproducible information about myocardial perfusion during stress [13]. This method allows for the assessment of perfusion defects, due to epicardial [19] or micro-vascular coronary artery disease [20,21]. Compared to other imaging modalities, stress CMR has no window or body habitus limitations and is the technique of choice for diagnosis of epicardial and micro-vascular CAD, mainly in those unable to exercise, but only if it is performed in experienced centers [13].
  **(e)**  **T1 mapping and ECV:** Although LGE is well established as the technique of choice for the detection of replacement fibrosis, it has inherent limitations to assess diffuse myocardial fibrosis, because it is based on changes in signal intensity between scarred and normal myocardium [13,21,22]. Therefore, parametric imaging including T1, T2 mapping, T2* and ECV, was developed. T1 mapping (native or pre-contrast T1 and post-contrast T1) provides a quantitative assessment of tissue T1 values and enables the identification of diffuse myocardial fibrosis, which is usually undetectable by the currently used blood biomarkers [22]. Normal values of T1 mapping are 995.8 ± 30.9 ms at 1.5 T [23] and 1183.8 ± 37.5 ms at 3T [24]. However, field strength and different types of pulse sequences influence T1 measurements. Therefore, it is recommended that different MRI units should generate their own normal values for use in clinical practice [21]. Post-contrast T1 mapping is used for ECV calculation in combination with native T1 mapping. ECV estimation requires measurement of myocardial and blood T1 before and after administration of contrast agents being determined as follows:
    $$\mathrm{ECV}=\left(1-\mathrm{Hematocrit}\right)\times \frac{\left(1/T1_{\left(\mathrm{myo}\;\mathrm{post}-\mathrm{contrast}\right)}\right)-\left(1/T1_{\left(\mathrm{myo}\;\mathrm{pre}-\mathrm{contrast}\right)}\right)}{\left(1/T1_{\left(\mathrm{blood}\;\mathrm{post}-\mathrm{contrast}\right)}-1/T1_{\left(\mathrm{blood}\;\mathrm{pre}-\mathrm{contrast}\right)}\right)}$$
    Normal ECV values of 25.3 ± 3.5% have been reported in healthy individuals at 1.5 T [21]. Apart from amyloid, increased ECV is most often due to excessive collagen deposition as in diffuse fibrosis accompanying systemic sclerosis [21] and other non-rheumatic processes. ECV is more reproducible than native and post-contrast T1 at different field strengths, vendors, and acquisition techniques [22,23].
    Increased native T1 mapping in the remote myocardial infarction area carries an ominous prognosis. Additionally, increased values of native T1 mapping and ECV may be an early finding in various cardiomyopathies, before the detection of strain, and strain rate abnormalities [24]. Furthermore, native T1 mapping is also sensitive to myocardial edema, iron overload and diffuse scarring [13], allowing for the monitoring of longitudinal changes associated with treatment in clinical trials [24]. Using these techniques, CMR can guide patient selection to revascularization in coronary chronic total occlusions [25].
**4.**  **Angiography using T1-W Imaging**
  Magnetic resonance angiography (MRA) is based on two general concepts:
  **(a)**  Methods relying on natural flow effects such as time-of-flight and phase-contrast techniques, either in two- or three-dimensional acquisition modes. Non-contrast MRA can provide pivotal information regarding large vessel aneurysm/stenosis without the need for contrast administration, while black blood images depicting increased wall thickness in a circumferential pattern characterize large vessel vasculitis [26].
  **(b)**  Contrast-enhanced (CE) MRA method, which is as accurate as X-ray angiography in detecting abnormalities of the great vessels, with important applications in rare autoimmune diseases with cardiovascular involvement, such as Takayasu arteritis, Behcet–Adamandiadis disease, Cogan disease and IgG4 arteritis [26]. Contrast-enhanced MRA is also frequently used to establish large vessel patency and identify mural inflammation in large vessel vasculitis [26]. The lack of radiation makes this technique ideal for serial evaluation.
    Additionally, we should mention that coronary artery vasculitis imaging is feasible using CMR methods presently at the investigational level. However, it is important in the evaluation of children with Kawasaki disease and coronary artery aneurysms (CAA) [27]. Moreover, stress CMR can assess myocardial ischemia in Kawasaki disease [28].
**5.**  **Pulmonary hypertension (PH) assessment**
  The International Guidelines recommended hemodynamic criteria for the diagnosis of PH include elevated mean pulmonary artery pressure (mPAP) of >20 mmHg with a pulmonary capillary wedge pressure (PCWP) ≤15 mmHg and a pulmonary vascular resistance (PVR) >3 Wood units (WU) [29]. Echocardiography remains the standard imaging modality for non-invasive estimation of PAP, with CMR playing an important complementary role [30,31], by providing unique structural/functional information on the pulmonary artery and RV, which have significant prognostic value for these patients. CMR SSFP allows for accurate quantification of RV mass, volumes, and wall motion abnormalities with high reproducibility [29]. LGE at the RV insertion point is commonly found in PH and is not indicative of disease severity [30]. A CMR model using interventricular septum angle, RV-LV mass ratio and PA anatomy, was found to have a sensitivity of 93% and specificity of 79% to detect PH non-invasively [32].
  Finally, four-dimensional flow (4D flow) is a new CMR method that allows 3D visualization of vascular flow and quantitative assessment of transvalvular or intra-cavity flow [33]. Abnormal flow patterns in the main pulmonary artery (MPA) are associated with PH and can be used to estimate mean pulmonary artery pressure (mPAP) and MPA wall shear stress, with reliable quantification of tricuspid regurgitation [33].
**6.**  **Valve heart disease (VHD) assessment**
  Echocardiography remains the main imaging modality used for diagnosis and long-term follow-up in RCD patients with valve heart disease. However, the low inter-study variability makes CMR an excellent alternative for serial assessment of VHD [34,35]. In patients with mitral regurgitation, total LV stroke volume is equivalent to the total aortic forward stroke volume (total anterograde flow) plus the mitral regurgitant volume (retrograde mitral flow) and can be accurately quantified by CMR.
  In aortic stenosis, phase-contrast velocity mapping can measure peak velocity across the valve. However, this approach is reserved for patients with poor echocardiographic windows because the lower temporal resolution of CMR, compared with Doppler echocardiography, may lead to an underestimation of disease severity. Aortic valve area (AVA) can be measured by CMR using planimetry [35], although such a technique remains inferior to AVA assessment by Doppler echocardiography. Conversely, the reproducibility of CMR in quantifying the severity of valvular regurgitation is superior to TTE and provides powerful prognostic information [36].
  **(a)**  **Iron deposition**
    Myocardial iron deposition in the heart cannot be predicted from serum ferritin or liver iron content in a biopsy. Furthermore, the conventional assessment of cardiac function can only detect patients with advanced iron cardiomyopathy. CMR is the only non-invasive imaging modality that can reproducibly quantify myocardial iron deposition using myocardial T2*. This CMR parameter is the most significant variable for predicting the need for iron chelation treatment. Early start or intensification of iron chelation treatment, guided by CMR, can reverse iron cardiomyopathy, and increase survival [37]. Finally, native T1 mapping can also measure iron and preliminary data show that it may have higher sensitivity in the detection of early iron overload [38].

## 3. CMR Limitations

1. Generally, the availability/expertise of CMR is rather low, but recently it has increased, as there is a great demand in cardiovascular clinical practice.
2. Contra-indicated in patients with metallic clips and non-MRI conditional devices [13]. However, even non-MRI conditional devices can be scanned if there is the expectation for a very important diagnostic benefit for the patient.
3. Gadolinium-based contrast agents (CA) should be used with caution in patients with impaired renal function (GFR < 30 mL/min) [13]. Importantly, several non-contrast sequences, such as SSFP, T2, native T1 mapping and ECV can provide valuable information in relevant cases [13].
4. The use of gadolinium contrast with MRI should be limited; it may be used as a contrast agent in pregnancy only if a significant improvement in diagnostic performance is expected with a serious impact on the fetal or maternal outcome. Breastfeeding should not be interrupted after gadolinium administration [39].
5. Traditionally, it is considered an expensive modality. However, there is a cost-minimization analysis for cardiac revascularization in CAD patients that showed the clear benefit of using CMR in decision making [40].

## 4. CMR for the Evaluation of Cardiovascular Involvement in RCDs

In **Class I**, patients with either genetic or autoimmune vascular diseases are included. In these patients, CMR can provide information on both heart and systemic circulation in the same examination. We note that various vascular diseases, such as Marfan syndrome, may affect both the heart and aorta, including mitral valve prolapse, heart failure, arrhythmias, and aortic dilatation/dissection [41]. CMR is more sensitive than an echo for identifying cases with mild systolic dysfunction, and strain analysis is more sensitive than a simple LVEF assessment for identifying Marfan patients with mild cardiomyopathy [42]. Furthermore, serial CMR measurements in children and young adults with connective tissue diseases (CTDs) and aortic stiffness progressively increase with age [43]. Finally, unenhanced SSFP CMR imaging allows for safe aortic monitoring with high diagnostic accuracy in patients with Marfan syndrome after aortic root surgery [44]. In Turner syndrome, CMR can identify more lesions than echocardiography, with aortic dilatation and bicuspid aortic valve being the most commonly missed lesions by this method [45].

**Figure 2:**
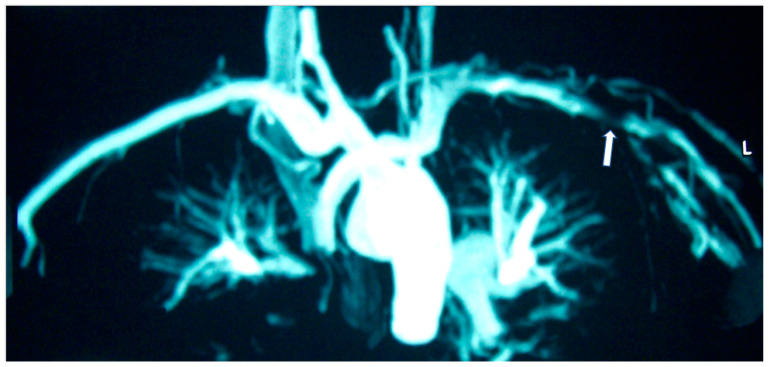
MRA of a patient with Takayasu disease showing left (L) subclavian artery stenosis (arrow).

In autoimmune vascular diseases, CMR can give valuable information about cardiac function, perfusion, type of fibrosis and vascular integrity that may significantly contribute to treatment decisions beyond vascular scores, other disease activity, severity indices or acute phase response [46]. In great vessel vasculitis, such as Takayasu disease (TA), CMR can provide valuable information about aortic and large vessel stenosis/myocardial inflammation and concurrent valvular disease (Figure 2) [47]. In medium vessel vasculitis, such as Kawasaki disease (KD), CMR offers important information during the acute and chronic phases of this disorder [48]. In the acute phase, it can identify myocardial inflammation, microvascular disease, myocardial infarction, heart failure, changes in the coronary artery lumen and changes in the coronary artery vessel wall. During the chronic phase, CMR may serve as a valuable tool for the detection of perfusion defects and risk stratification/treatment guide [48]. Finally, in small vessel vasculitis, such as Churg–Strauss syndrome, CMR can detect cardiac involvement either in the form of diffuse subendocardial fibrosis (DSF) or myocardial inflammation in both ANCA (+) and ANCA (-) CSS, although it is clinically overt in ANCA (-). DSF carries an ominous prognosis for LV function [49].

In **Class II**, which includes rare pulmonary circulation diseases, both the pulmonary valve and trunk with its main branches can be hardly visualized using echo, with most abnormalities about pulmonary circulation coming from autopsy data. CMR is an excellent tool for the visualization of both pulmonary valves and arteries. In Class II, patients with pulmonary vessels abnormalities, CMR can identify the abnormal origin of the left coronary artery from the pulmonary artery [50], a quadricuspid pulmonary valve and left pulmonary artery aneurysm [51], and pulmonary valve regurgitation in patients with repaired Fallot tetralogy [52]. Furthermore, ventricular function and myocardial scar before and early after repair of an anomalous left coronary artery from the pulmonary artery and/or Fallot tetralogy can be successfully assessed by CMR [53].

CMR can be used for the evaluation of cardiovascular function/morphology and haemodynamics relevant to pulmonary artery hypertension (PAH) [54]. CMR is considered a powerful prognostic marker with impaired RV function and increased RV-LV volumes predicting clinical worsening and mortality [55]. There is good agreement in flow quantification between mPAP estimated by CMR and TRPG estimated by echocardiography. 

Finally, CMR 4D flow has higher diagnostic power for detecting increased PA pressure, than echocardiography. This is due to (a) the lower sensitivity of echo in detecting increased PA pressure compared with CMR, and (b) limitations in the visualization/measurement of the TR jet by echo [56].

In **Class III,** which includes various cardiomyopathies, CMR is the sine qua non modality for diagnosis of preclinical/clinical disease and treatment evaluation [57]. In patients with dilated cardiomyopathy (DCM), CMR can provide functional and tissue characterization in the same examination [57]. The LGE pattern in dilated cardiomyopathy should be discussed: DCM LGE patterns (1) no enhancement 60%, (2) subendocardial or transmural or (3) patchy or longitudinal striae of midwall (28% midwall linear) [58]. Furthermore, the presence of LGE indicates a high risk for adverse cardiovascular events in DCM patients [59].

In familial DCM with early conduction disease, such as Lamins A and C, which are intermediate filament nuclear envelope proteins encoded by the *LMNA* gene [60], the pretest probability of finding an LMNA mutation is high, and therefore, the early diagnosis of lamin A/C heart disease is important to plan early cardioverter defibrillator implantation.

**Figure 3:**
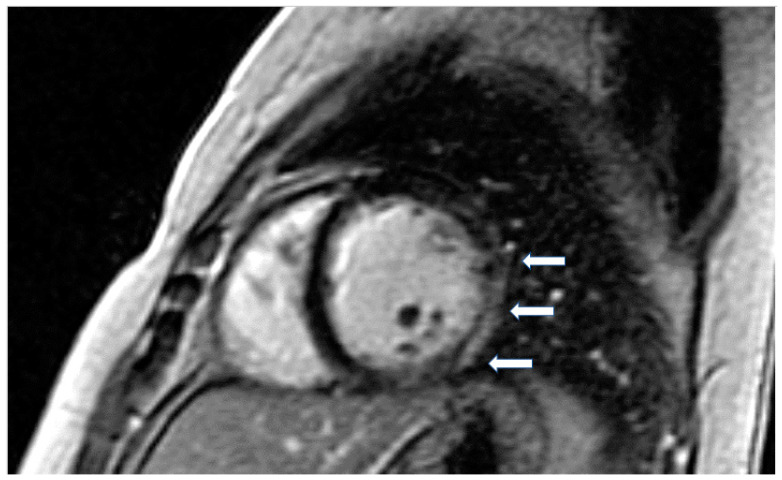
Short axis LGE image showing diffuse subendocardial fibrosis (arrows) in a patient with Churg–Strauss vasculitis.

According to previous studies, patients with LMNA had LV myocardial fibrosis in 88% of cases and LGE was associated with conduction abnormalities. In 69% of asymptomatic or mildly symptomatic patients, mild ventricular dilatation, systolic dysfunction, or both, was found associated with decreased longitudinal systolic LV function in 53% of them [61]. Finally, in dystrophinopathies, CMR has documented a pattern of epicardial fibrosis in the inferolateral wall of LV in both patients and carriers (Figure 3). This can be observed even if the overt muscular disease is absent. Recently, CMR parametric techniques have been used in Duchenne muscular dystrophy to detect diffuse myocardial fibrosis [62].

Myocardial hypertrophy is a common characteristic in various cardiomyopathies, including hypertensive (HTN), hypertrophic (HCM), aortic stenosis (AS)-related and, chronic kidney disease cardiomyopathy (CKD), as well as amyloidosis (CA), athletes’ heart (AH) and Anderson–Fabry disease (AFD). CMR allows for the detailed characterization of the hypertrophic phenotype, which makes it possible to differentiate HCM from other causes of LV hypertrophy. Due to its high contrast between the blood pool and the myocardium, SSFP cine imaging is used for morphological assessment and to quantify ventricular volumes, ejection fraction, and mass, which has prognostic implications. Cine SSFP can also demonstrate the presence of a turbulence jet across the left ventricular outflow tract (LVOT) in patients with obstructive HCM, and aid in the exact location of the flow obstruction site. Moreover, SSFP CMR can detect other abnormalities associated with HCM such as the presence of congenital ventricular outpouchings (recesses, diverticula, aneurysms, clefts, and crypts), anomalies in the mitral valve apparatus and abnormalities of the papillary muscles. It may also facilitate the diagnosis of apical HCM, frequently missed by echocardiography [63].

**Figure 4:**
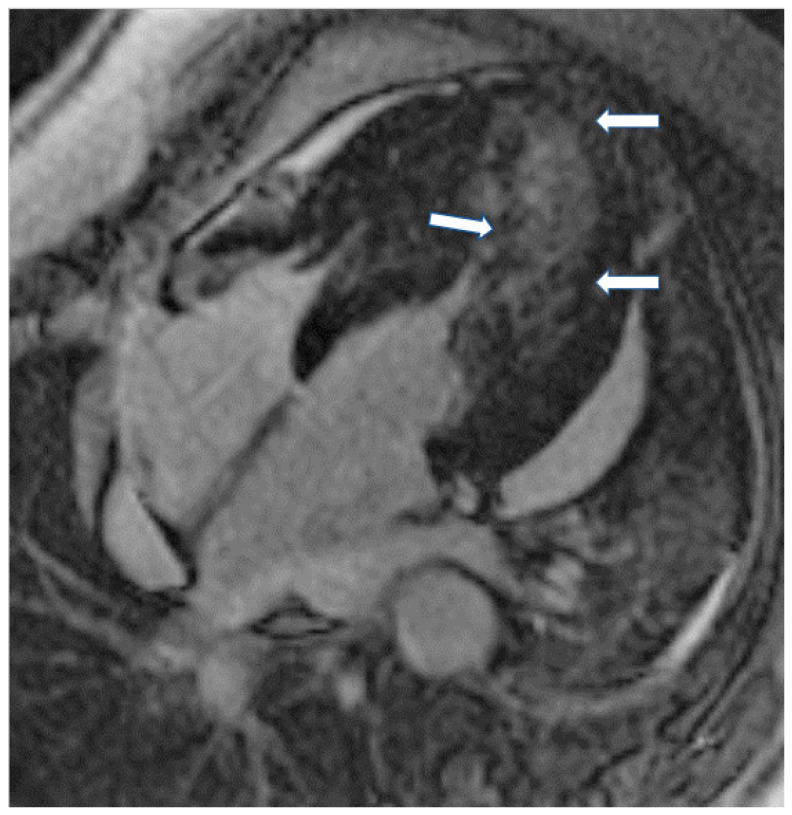
Four chamber inversion recovery image showing severe hypertrophy and extensive LGE (arrows).

The presence of LGE in HCM, usually in the hypertrophic area, is a high-risk feature, and its presence should be used as a marker for major adverse outcomes such as sudden cardiac death (SCD), arrhythmias, and failure (HF) (Figure 4). It should be also included as an additional index in decision making for implantable cardioverter defibrillators for primary prevention [64]. LGE is usually present in segments with hypertrophy, in some end-stage cases, some segments may appear thinned with transmural fibrosis. The extent of LGE can be quantified either as a sum of the enhanced areas measured in grams or as a proportion of the total left ventricular mass (percentage of LGE). The percentage of fibrosis varies according to the quantification method used. From those methods, the only validated method against necropsy is the semi-automatic 2-standard-deviation technique, which consists of defining LGE as a 2-standard deviation above the mean signal intensity of the distant myocardium and constitutes the preferred quantification method. LGE is rarely observed in mutation carriers without LVH. In a study including patients with pathogenic sarcomere mutations and hypertrophic cardiomyopathy, subjects with mutations but no LV hypertrophy, and controls, CMR showed LGE in 71% of subjects with overt hypertrophy but in none of the mutation carriers without hypertrophy. Different studies have shown an increase in the risk of ventricular arrhythmias in patients with HCM related to the presence of fibrosis evaluated by LGE in comparison with individuals without LGE [63].

In various types of myocardial hypertrophy, including chronic kidney disease (CKD), hypertension (HTN), and hypertrophic cardiomyopathy (HCM), a different CMR signature of hypertrophic phenotypes was recently identified. Native T1 was raised in all conditions, indicating pathologic hypertrophic remodeling. Markedly raised native T2 was CKD-specific, suggesting a prominent role of intramyocardial edema [65]. In contrast to HCM, LGE in AFD is usually located in the lateral wall of the LV. Furthermore, a native T1 mapping reduction even in cases before the onset of LVH in AFD is associated with early diastolic and systolic changes measured by echo and represents a typical characteristic of the disease [66]. The International T1 Multicenter Cardiovascular Magnetic Resonance Study showed that native T1 and ECV were significantly higher in HCM compared with hypertensive patients, even when including HCM patients without LGE and hypertensive subjects with LV wall thickness of >15 mm [67].

Restrictive cardiomyopathies are the least common forms of heart muscle disease. They may be infiltrative or non-infiltrative and related to storage diseases and endomyocardial disorders. Although genetic diseases commonly present during childhood or adolescence, there is a growing number of older patients with heart failure and preserved ejection fraction (HFpEF) diagnosed as having restrictive cardiomyopathy, particularly cardiac amyloidosis. The detection of infiltrative cardiomyopathies, particularly primary and secondary forms of iron overload, as well as inflammatory diseases, such as sarcoidosis, has slowly led to improved outcomes via disease-specific therapies [68].

CMR is crucial for the assessment of restrictive cardiomyopathies (RCM). In the case of iron overload, it is the only available non-invasive modality that can assess quantitatively iron in various organs, including the heart [69]. Iron’s para-magnetic effect produces changes in the magnetic resonance signal intensity, shortens the T2* relaxation time and darkens the image more quickly [69]. T2* relaxation time is an excellent measure of myocardial iron deposition and is useful for serial assessment of response to iron chelation therapy. Decreasing T2* values, are correlated with increasing iron deposition and inversely correlated with global cardiac function. In RCM due to iron overload, T2* values are typically <20 ms. A T2* value < 10 ms is indicative of severe iron overload and predicts subsequent risk of heart failure with a sensitivity of 97.5% and specificity of 83% [69]. T2* imaging has also been shown to be highly predictive of subsequent arrhythmia development [69]. We also note that in established cardiac iron overload, T1 mapping and T2* are concordant. However, in the 20–30 ms T2* range, T1 mapping appears to detect iron. These data support previous suggestions that T1 detects missed iron in 1 out of 3 subjects with normal T2*, and that T1 mapping is complementary to T2* [38].

In patients with endomyocardial fibrosis, CMR can provide additional information regarding chamber distortion and LV cavitary thrombus burden. Furthermore, LGE correlates well with the extent of fibrosis [70].

Finally, CMR is increasingly utilized in the evaluation of silent cardiac sarcoidosis (CS), due to its ability to identify small areas of myocardial involvement in patients with preserved systolic function or early stage CS [71]. Increased T2 mapping denotes acute myocardial inflammation, due to edema, while the presence of LGE denotes myocardial scar. In addition to its diagnostic role, CMR has also an increasing role in predicting adverse cardiovascular events [72]. The extent of LGE correlates well with the severity of cardiac involvement, due to both acute inflammation and cardiac fibrosis [72]. Patients with a significant amount of LGE have an increased risk of life-threatening ventricular arrhythmias and SCD, with a reported hazard ratio of 31.6 for lethal events and 33.9 for all adverse cardiac events, independent of ejection fraction or LV volume and supports the insertion of implantable defibrillator [73]. Furthermore, LGE has also been shown to predict corticosteroid responsiveness, with a large LGE being associated with a lack of systolic function improvement [74].

The manifestations of radiation-induced heart disease (RIHD) include accelerated coronary artery disease (CAD), valvular dysfunction, RCM, aortopathy, and constrictive pericarditis [75]. RICM is characterized by inflammation followed by the development of a diffuse, patchy interstitial fibrosis of the myocardium, which is usually detected either by EMB or at autopsy. Although CMR rarely reveals the presence of LGE in RIHD, native and post-contrast T1 mapping by CMR enables estimation of extracellular volume (ECV), which is increased as a result of diffuse myocardial fibrosis. The assessment of myocardial fibrosis using ECV should be used for early detection of myocardial lesions post-radiation treatment [76].

**Figure 5:**
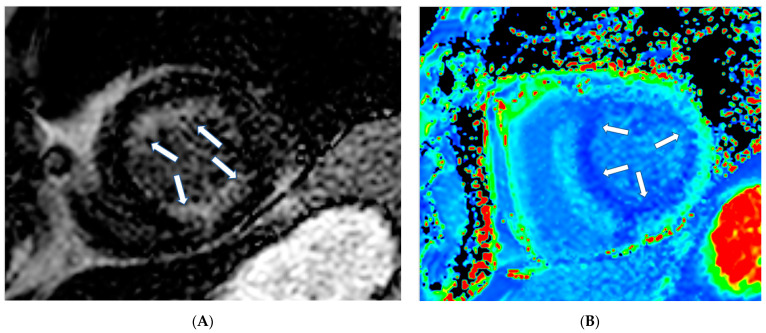
(**A**) Short axis LGE image showing diffuse amyloidosis (arrows). (**B**) Matching native T1 mapping of the same patient.

Finally, cardiac amyloidosis (CA) (Figure 5A,B), Takotsubo cardiomyopathy (TCM), non-compaction cardiomyopathy (LVNC), and cardiomyopathies associated with neuromuscular, metabolic, and autoimmune diseases are included in the unclassified cardiomyopathies.

In CA, CMR shows diffuse hypertrophy of both LV-RV, in contrast to HCM, which is usually associated with focal hypertrophy. Thickening of the interatrial septum and posterior right atrial wall >6 mm is also common in CA [77]. LGE is the hallmark of cardiac amyloidosis on CMR. In a study by Vogelsberg et al. [78], LGE was found in 79% of patients with CA. LGE may involve the entire subendocardium, extending into the neighboring myocardium. Ejection fractions, LV end-diastolic volume and myocardial mass were not significantly different between the CA group and the other groups with various cardiac disorders. In this study, the average interventricular septum was 17 ± 4 mm in the CA group compared with 13 ± 3 mm in the non-CA group [78]. A more recent study by Syed et al. [79] showed that CMR demonstrated LGE in 97% of patients and increased LV wall thickness in 91%. We should also note that LV amyloid accumulations occur according to the following steps: (a) no evidence of LGE, (b) appearance of subendocardial LGE (typical pattern), and (c) progression to transmural LGE [80].

We should emphasize that (a) CMR cannot differentiate the various causes of amyloidosis and (b) the nulling pattern of myocardium and blood pool in CA shows temporal variability with earlier onset of reverse nulling pattern in time of inversion scout (TIS) sequence showing a trend toward more LVM and possibly more severe amyloid load. Although TIS images are advisable at multiple time points, 10 min or later images rather than the earlier images would be more useful to diagnose CA based on reversed nulling pattern (RNP) [81].

Finally, patients with CA presented with pleural effusions that were associated with poor RV function in AL. However, such an association was not identified with pericardial effusion [82].

In TCM, CMR shows apical dilatation, extensive edema in STIRT2 images, increased values of native T1, and T2 mapping, and absence of LGE in inversion recovery images in the majority of cases [83]. However, if LGE is present, it is associated with adverse outcomes. [84].

LVNC, also known as spongiform cardiomyopathy, is a phenotype of hypertrophic ventricular trabeculations with deep interventricular recesses. CMR has a better contrast resolution compared with echo and is considered as the modality of choice for the diagnosis of LVNC. The best diagnostic criterion is a ratio of non-compacted end-diastolic to compacted end-diastolic myocardium of more than 2.3:1 (sensitivity: 86%, specificity: 99%). For improved discrimination of LVNC versus other cardiomyopathies with hypetrabeculated myocardium, the following MRI criteria have been proposed in a recent study [85]:

(A) percentage LV myocardial mass (non-compacted) > 25%;
(B) total LV myocardial mass index (non-compacted) > 15 g/m^2^;
(C) non-compacted/compacted myocardium ratio of ≥3:1 in at least one of the following segments (1–3, 7–16)—the apical segment 17 is excluded;
(D) trabeculation (non-compacted/compacted) in segments 4–6 of ≥2:1.

In cardiomyopathies due to neuromuscular disorders, hypertrophic phenotypes can be observed in Friedreich ataxia, Barth syndrome, some forms of Duchenne/Becker disease, a dilated phenotype of Duchenne/Becker disease, myotonic dystrophy, and Limb-girdle muscular dystrophy [86]. CMR can assess biventricular function and the presence of replacement fibrosis presenting as LGE that is patchy in the hypertrophic area in those with hypertrophic phenotype and subepicardial in the lateral wall of LV in those with dilated phenotype [87].

Metabolic disorders, such as thyroid disease, pheochromocytoma and growth hormone excess or deficiency, may contribute to usually reversible dilated cardiomyopathy. Glycogen storage diseases present myopathy, liver, and heart failure. Lysosomal storage diseases can provoke cardiac hypertrophy, mimicking hypertrophic cardiomyopathy and arrhythmias. Hereditary hemochromatosis, an inherited disorder of iron metabolism, leads to tissue iron overload in different organs, including the heart. Finally, nutritional disturbances and metabolic diseases, such as Kwashiorkor, Beri-Beri, obesity, alcohol consumption and diabetes mellitus may also lead to severe cardiac dysfunction that can be detected early by CMR (increased T1 mapping and ECV before the development of LV dysfunction) [88,89].

Cardiovascular disease (CVD) is a major cause of mortality and morbidity in patients with chronic inflammatory disorders, such as rheumatoid arthritis (RA) and other seronegative arthritis, systemic lupus erythematosus (SLE), antiphospholipid syndrome, systemic sclerosis (SSc), chronic human immunodeficiency virus (HIV) infection, and psoriasis. Patients with chronic inflammatory diseases have an increased risk of coronary heart disease (CHD), stroke, peripheral vascular disease (PVD), and cardiomyopathy that may not be fully captured by traditional CVD risk factors, such as dyslipidemia, aging, hypertension, and smoking [90].

In autoimmune rheumatic diseases (ARDs), CMR can be used for evaluating cardiac anatomy, mass, wall motion, and atrial/ventricular function. T2-weighted imaging (T2-W) can be used for edema detection, which appears as a high signal intensity area on STIRT2 (short tau inversion recovery) images. T2 mapping, a newer T2-W technique, can provide a better assessment of myocardial edema. The positivity of T2 imaging is indicative of an acute/recent myocardial lesion. Lastly, LGE images, allow the detection of myocardial replacement but not of diffuse myocardial fibrosis. In contrast to LGE, native T1 mapping and extracellular volume fraction (ECV) can reliably quantify diffuse myocardial fibrosis. The typical patterns of fibrosis in ARDs include subepicardial patchy fibrosis usually in the inferolateral wall of LV, in the presence of active or past myocarditis, subendocardial/transmural fibrosis following the distribution of coronary arteries in CAD, diffuse subendocardial fibrosis in small vessel vasculitis, systemic sclerosis, and anti-phospholipid syndrome [90].

**Class IV** includes rare congenital cardiovascular diseases which may affect valvular anatomy/function, cardiac muscle abnormalities and/or vascular abnormalities. CMR can provide an angiographic assessment of great vessels/ shunts/ Qp-Qs and information regarding the anatomy/function of ventricles/atria, and diffuse or replacement fibrosis that is of great value for decision making in these patients [91].

**Class V** includes oncologic patients. Myxomas represent the majority of benign tumors, while sarcomas are the most common primary malignant tumors [92,93]. Metastatic malignant tumors of the heart and pericardium are more common than primary cardiac tumors. Cardiac involvement is as high as 20% in patients with diagnosed cancer in other organs [94].

**Figure 6:**
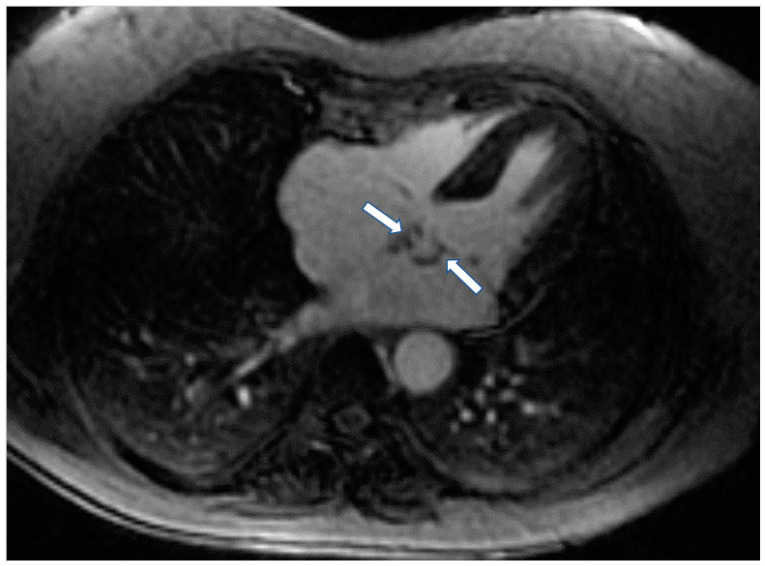
Four chamber inversion recovery image showing the presence of fibroma (arrows) in left atrium.

The high spatial resolution of CMR, in parallel with tissue characterization, helps to assess tissue invasion and differentiate potential etiologies. Mass localization is performed with dark- and white-blood imaging, followed by tissue characterization based on T1- and T2-weighted imaging, fat, and water suppression sequences, first-pass perfusion, early (EGE) and LGE (Figure 6) [95].

**Figure 7:**
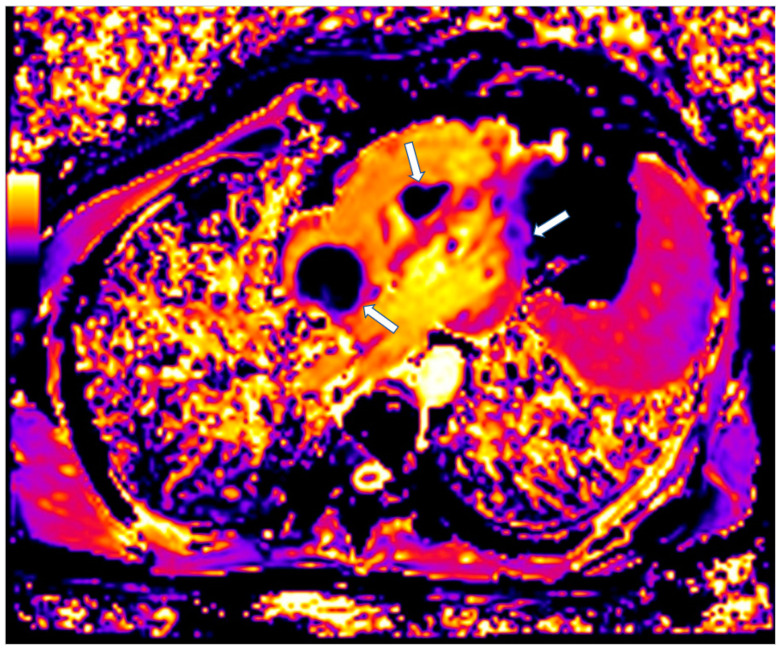
Four chamber T1 mapping showing LV infiltration and RV-Right atrium masses (arrows) due to melanoma.

CMR is also of value in the detection of intracardiac thrombi. Usually, the majority of thrombi is small, homogeneous, often associated with venous catheters and typically hypointense on first-pass perfusion, due to lack of vasculature [96]. Compared to thrombi, malignant tumors tend to be larger with first-pass perfusion abnormalities and LGE [96,97]. Parametric mapping has also demonstrated important diagnostic value (Figure 7) [97].

Finally, CMR is of great value in the evaluation of cardiotoxic effects, due to chemotherapy used in various oncologic diseases. In this context, early detection of cardiotoxicity is crucial and presents an opportunity for personalized risk stratification and early therapeutic intervention before irreversible heart failure occurs. CMR is superior to echocardiography for detecting subclinical declines in LVEF early after cardiotoxic therapy. Even when applying 3D-TTE techniques, which demonstrated less variability, this methodology lacked the desired accuracy to reliably identify subtle 5% changes in LVEF that may have major management implications [98].

Tissue characterization using parametric pixel-wise T1 and T2 mapping is extremely useful in detecting subtle myocardial inflammatory changes and has been validated in a variety of systemic conditions, including the diagnosis of myocarditis. Additionally, the measurement of ECV has also shown significant promise as a surrogate marker for diffuse myocardial fibrosis, overcoming the limitations of LGE in this regard. An expanded ECV is associated with adverse outcomes in large patient cohorts [99].

Finally, immune checkpoint inhibitor (ICI)-induced cardiotoxicity is a rare immune-related adverse event (irAE) characterized by a high mortality rate. The diagnosis of ICI-induced cardiotoxicity can be challenging, and CMR is the diagnostic tool of choice in clinically stable patients with suspected myocarditis [100].

**Class VI** includes cardiac arrhythmogenic disorders.

Arrhythmogenic cardiomyopathy (ACM) is an emerging new concept of a life-threatening heart muscle disorder, due not only to desmosome gene mutations, but also to non-desmosome genes, such as filamin C, lamin A/C, phospholamban, transmembrane protein 43, titin, SCN5A, and RNA binding motif protein 20. CMR is the gold standard for evaluating left and right ventricular structure/function, edema, and fibrosis. The identification of regional fibrosis with LGE has prognostic value. In ACM patients with only mild LV dysfunction but with evidence of phospholamban, filamin C or lamin A/C mutations, an ICD is considered a reasonable approach [101].

Fibro-fatty myocardial replacement is the diagnostic criterion of arrhythmogenic cardiomyopathy (ACM) that can affect both RV-LV. The 2010 International Task Force diagnostic criteria lacked specific criteria for the diagnosis of LV variants of ACM [102].

The current classification of ACM according to Padua criteria includes the following phenotypic variants: (i) the *“dominant-right”* variant, i.e., the classic ARVC phenotype characterized by the predominant RV involvement, with no or minor LV abnormalities; (ii) the *“biventricular disease”* variant characterized by the parallel involvement of the RV and LV; and (iii) the *“dominant-left”* variant (also referred to as “arrhythmogenic left ventricular cardiomyopathy; ALVC”) characterized by the predominant LV involvement, with no or minor RV abnormalities. ALVC is a very rare non-ischemic cardiomyopathy. CMR shows that ALVC presents fibro-fatty replacement predominantly in the LV, impaired left ventricular systolic function and ventricular arrhythmias originating from the LV. ALVC with RV involvement has a worse prognosis [103]. We should also notice that among patients with definite arrhythmogenic right ventricular cardiomyopathy (ARVC), patients with normal CMR have a low risk of arrhythmic events and do not require ICD, while patients with ALVC involvement are at particularly high risk and require ICD [103]. In contrast, patients with isolated RV involvement have an intermediate risk of arrhythmic events and can be further risk stratified using the ARVC score including age at diagnosis, gender, presence of cardiac syncope, number of inverted T waves in precordial and inferior leads, maximum 24 h premature ventricular contraction count, history of non-sustained VT and the RV ejection fraction [104].

The Padua criteria for diagnosis of ACM are based on a multi-parametric approach encompassing functional and structural ventricular abnormalities, tissue characterization findings, depolarization and repolarization electrocardiographic alterations and ventricular arrhythmias, and familial/genetic background which are grouped into 6 categories. The novelty of the Padua diagnostic criteria essentially consists of the introduction of tissue characterization by contrast-enhanced (CE-CMR) for the detection of fibro-fatty myocardial replacement of both ventricles and the addition of new ECG criteria, including depolarization/ repolarization abnormalities and ventricular arrhythmias, specific for the LV involvement.

**Class VII** includes rare CVDs and disorders in pregnancy. Peripartum cardiomyopathy develops most frequently in the month pre- or post-partum, whereas DCM often is known already or develops in the second trimester. Mortality in peripartum cardiomyopathy varies from <2% to 50%. There are few reports on DCM and pregnancy, including only a limited number of patients. Ventricular arrhythmias, heart failure, stroke, and death occur in 39–60% of high-risk patients. However, patients with modest LV dysfunction tolerated pregnancy well. Previous studies on >700 pregnancies in 500 women with HCM showed a good prognosis, even though three deaths were reported in high-risk patients [104]. Complications include supra-ventricular and ventricular arrhythmias, heart failure, and ischemic stroke. Recent studies on 200 pregnancies in 100 women with ARVC have reported symptoms, including heart failure in 18–33% of pregnancies. Ventricular tachycardia was found in 0–33% of them, and syncope occurred in one patient [105].

Although echo is the first-line modality for assessing maternal cardiac status, CMR can provide information about cardiac anatomy/function in pregnant women with complex cardiac disease or suspected aortic pathology. Furthermore, LV remodeling during normal pregnancy is associated with myocardial hypertrophy without edema or diffuse fibrosis of the myocardium, or LV systolic dysfunction. These findings, observed in normal pregnancy, can serve as an important basis for identifying myocardial abnormalities in patients with peripartum cardiomyopathy and other pregnancy-related myocardial diseases [106].

**Class VIII** includes unclassified rare cardiovascular diseases/disorders, such as isolated ventricular non-compaction (IVNC), characterized by prominent intra-ventricular trabeculations separated by deep intertrabecular recesses. Although microvascular dysfunction is known, myocardial infarction is rare and usually seen as a consequence of coincidental coronary artery disease [107]. In these cases, CMR can have an important place in identifying anatomy, function, and perfusion defects [108].

## 5. Take Home Messages

1. CMR can perform angiography, function, perfusion, and tissue characterization in the same examination;
2. Evidence of edema expressed as a high signal in STIRT2 or increased absolute T2 values on T2 mapping is common in acute/active inflammatory states;
3. Diffuse subendocardial fibrosis, expressed as diffuse, subendocardial LGE, is characteristic of microvascular disease, as in systemic sclerosis, small vessel vasculitis, anti-phospholipid syndrome, cardiac amyloidosis, and cardiac involvement in metabolic disorders;
4. Replacement fibrosis, expressed as LGE, in the inferolateral wall of LV is common in neuromuscular disorders, such as DMD and BMD. However, other myocardial areas can be also involved;
5. Active sarcoidosis can present with punched-out LGE with edema, irrespective of the cause and blood biomarkers;
6. Cardiac hypertrophy is characteristic in HCM, CA and AFD, but LGE may be located in IVS, subendocardium and lateral wall in HCM, CA and AFD, respectively. Furthermore, increased absolute T1 values or T1 signal on T1 mapping are characteristic of HCM, CA, while reduced T1 signal on T1 mapping is characteristic of AFD;
7. Magnetic resonance angiography provides non-invasive information in various aortopathies, such as in Marfan, Turner syndromes and Takayasu, giant cell, Cogan and Behcet vasculitis;
8. LGE in RV is the typical finding of ARVC. However, LGE can be also found in LV leading to the diagnosis of ACM, according to new Padua criteria;
9. The tissue changes in RCDs may be detected only through changes in parametric imaging indices, which include native T1, T2 mapping and ECV, while all other indices may be normal.

## 6. Conclusions

RCDs have a low incidence, but their impact on the survival and quality of life of the affected patients and their families is of great importance. Recently, the new classification of RCDs allowed a better understanding of their pathophysiology. Cardiovascular imaging, and specifically CMR, plays a pivotal role in the diagnosis and risk stratification of these patients.

CMR, due to its capability to perform anatomy, function, perfusion, angiography, and tissue characterization, is the ideal tool to screen RCDs and identify even the most subtle, subclinical changes in this special group of patients.

## Figures and Tables

**Table 1:** Clinical classification of rare cardiovascular diseases/disorders.

**Class I**	**Rare Diseases of Systemic Circulation**
Group 1	Anatomical malformations of the arteries
Group 2	Connective tissue disorders causing aneurysmal disease
Group 3	Autoimmune vascular diseases
Group 4	Intimal hyperplasia
Group 5	Spontaneous dissection of the artery
Group 6	Premature atherosclerosis
Group 7	Others
**Class II**	**Rare diseases of pulmonary circulation**
Group 1	Pulmonary hypertension
Group 2	Inborn anomalies of the pulmonary vessels
Group 3	Acquired anomalies of the pulmonary vessels
**Class III**	**Rare diseases of the heart (cardiomyopathies)**
Group 1	Dilated cardiomyopathy
Group 2	Hypertrophic cardiomyopathy
Group 3	Restrictive cardiomyopathy
Group 4	Arrhythmogenic right ventricular cardiomyopathy
Group 5	Unclassified cardiomyopathies
**Class IV**	**Rare congenital cardiovascular diseases**
Group 1	Abnormalities of the position and connection of the heart and vessels
Group 2	Shunts
Group 3	Complex congenital cardiovascular diseases
Group 4	Congenital cardiovascular diseases and concomitant organ dysfunction
Group 5	Grown-up congenital cardiovascular diseases
Group 6	Others
**Class V**	**Cardiac tumors and cardiovascular diseases in malignancy**
Group 1	Primary cardiac tumors
Group 2	Metastatic cardiac tumors
Group 3	Inflammatory malformations
Group 4	Cardiovascular complications of oncological therapy
**Class VI**	**Cardiac arrhythmogenic disorders and arrhythmias**
Group 1	Primary electrical disorders of the heart
Group 2	Arrhythmias in specific clinical settings
**Class VII**	**Rare cardiovascular diseases and disorders in pregnancy**
**Class VIII**	**Unclassified rare cardiovascular diseases/disorders**

## Data Availability

Not applicable.

## References

[1] McAllister A.K. (2002). BDNF. Curr. Biol..

[2] (2019). Inserm, Us14. Orphanet: About Rare Diseases. https://www.orpha.net/.

[3] Podolec P. (2013). Classification of Rare Cardiovascular Diseases (RCD Classification), Krakow 2013. J. Rare Cardiovasc. Dis..

[4] Mavrogeni S., Pepe A., Nijveldt R., Ntusi N., Sierra-Galan L.M., Bratis K., Wei J., Mukherjee M., Markousis-Mavrogenis G., Gargani L. (2022). Cardiovascular magnetic resonance in autoimmune rheumatic diseases: A clinical consensus document by the European Association of Cardiovascular Imaging. Eur. Heart J. Cardiovasc. Imaging.

[5] Liu C., Ferrari V.A., Han Y. (2021). Cardiovascular Magnetic Resonance Imaging and Heart Failure. Curr. Cardiol. Rep..

[6] Alraies M.C., AlJaroudi W., Yarmohammadi H., Yingchoncharoen T., Schuster A., Senapati A., Tariq M., Kwon D., Griffin B.P., Klein A.L. (2015). Usefulness of cardiac magnetic resonance-guided management in patients with recurrent pericarditis. Am. J. Cardiol..

[7] Rosmini S., Seraphim A., Captur G., Gomes A.C., Zemrak F., Treibel T.A., Cash L., Culotta V., O”mahony C., Kellman P. (2019). 247Characterisation of pleural and pericardial effusions with T1 mapping. Eur. Heart J. Cardiovasc. Imaging.

[8] Cosyns B., Plein S., Nihoyanopoulos P., Smiseth O., Achenbach S., Andrade M.J., Pepi M., Ristic A., Imazio M., Paelinck B. (2015). European Association of Cardiovascular Imaging (EACVI) position paper: Multimodality imaging in pericardial disease. Eur. Heart J. Cardiovasc. Imaging.

[9] Geske J.B., Anavekar N.S., Nishimura R.A., Oh J.K., Gersh B.J. (2016). Differentiation of Constriction and Restriction: Complex Cardiovascular Hemodynamics. J. Am. Coll. Cardiol..

[10] Francone M., Dymarkowski S., Kalantzi M., Rademakers F.E., Bogaert J. (2006). Assessment of ventricular coupling with real-time cine MRI and its value to differentiate constrictive pericarditis from restrictive cardiomyopathy. Eur. Radiol..

[11] Vidalakis E., Kolentinis M., Gawor M., Vasquez M., Nagel E. (2020). CMR in Pericardial Diseases—An Update. Curr. Cardiovasc. Imaging Rep..

[12] Glower D.D. (2016). Sticking points in magnetic resonance diagnosis of constrictive pericarditis. J. Thorac. Cardiovasc. Surg..

[13] Mavrogeni S.I., Kitas G.D., Dimitroulas T., Sfikakis P.P., Seo P., Gabriel S., Patel A.R., Gargani L., Bombardieri S., Matucci-Cerinic M. (2016). Cardiovascular magnetic resonance in rheumatology: Current status and recommendations for use. Int. J. Cardiol..

[14] Srichai M.B., Junor C., Rodriguez L.L., Stillman A.E., Grimm R.A., Lieber M.L., Weaver J.A., Smedira N.G., White R.D. (2006). Clinical, imaging, and pathological characteristics of left ventricular thrombus: A comparison of contrast-enhanced magnetic resonance imaging, transthoracic echocardiography, and transesophageal echocardiography with surgical or pathological validation. Am. Heart J..

[15] Rosmini S., Seraphim A., Knott K., Brown J.T., Knight D.S., Zaman S., Cole G., Sado D., Captur G., Gomes A.C. (2021). Non-invasive characterization of pleural and pericardial effusions using T1 mapping by magnetic resonance imaging. Eur. Heart J. Cardiovasc. Imaging.

[16] Giri S., Shah S., Xue H., Chung Y.C., Pennell M.L., Guehring J., Zuehlsdorff S., Raman S.V., Simonetti O.P. (2012). Myocardial T_2_ mapping with respiratory navigator and automatic nonrigid motion correction. Magn. Reson. Med..

[17] Wassmuth R., Prothmann M., Utz W., Dieringer M., von Knobelsdorff-Brenkenhoff F., Greiser A., Schulz-Menger J. (2013). Variability and homogeneity of cardiovascular magnetic resonance myocardial T2-mapping in volunteers compared to patients with edema. J. Cardiovasc. Magn. Reson. Off. J. Soc. Cardiovasc. Magn. Reson..

[18] Mahrholdt H.G.S., Schulz-Menger J., Schwitter J. (2020). CMR in Myocardits in: CMR-Update.

[19] Patel A.R., Salerno M., Kwong R.Y., Singh A., Heydari B., Kramer C.M. (2021). Stress Cardiac Magnetic Resonance Myocardial Perfusion Imaging: JACC Review Topic of the Week. J. Am. Coll. Cardiol..

[20] Levelt E., Piechnik S.K., Liu A., Wijesurendra R.S., Mahmod M., Ariga R., Francis J.M., Greiser A., Clarke K., Neubauer S. (2017). Adenosine stress CMR T1-mapping detects early microvascular dysfunction in patients with type 2 diabetes mellitus without obstructive coronary artery disease. J. Cardiovasc. Magn. Reson. Off. J. Soc. Cardiovasc. Magn. Reson..

[21] Moon J.C., Messroghli D.R., Kellman P., Piechnik S.K., Robson M.D., Ugander M., Gatehouse P.D., Arai A.E., Friedrich M.G., Neubauer S. (2013). Myocardial T1 mapping and extracellular volume quantification: A Society for Cardiovascular Magnetic Resonance (SCMR) and CMR Working Group of the European Society of Cardiology consensus statement. J. Cardiovasc. Magn. Reson. Off. J. Soc. Cardiovasc. Magn. Reson..

[22] Sibley C.T., Noureldin R.A., Gai N., Nacif M.S., Liu S., Turkbey E.B., Mudd J.O., van der Geest R.J., Lima J.A., Halushka M.K. (2012). T1 Mapping in cardiomyopathy at cardiac MR: Comparison with endomyocardial biopsy. Radiology.

[23] Barison A., Gargani L., De Marchi D., Aquaro G.D., Guiducci S., Picano E., Cerinic M.M., Pingitore A. (2015). Early myocardial and skeletal muscle interstitial remodelling in systemic sclerosis: Insights from extracellular volume quantification using cardiovascular magnetic resonance. Eur. Heart J. Cardiovasc. Imaging.

[24] Cao Y., Zeng W., Cui Y., Kong X., Wang M., Yu J., Zhang S., Song J., Yan X., Greiser A. (2018). Increased myocardial extracellular volume assessed by cardiovascular magnetic resonance T1 mapping and its determinants in type 2 diabetes mellitus patients with normal myocardial systolic strain. Cardiovasc. Diabetol..

[25] Pica S., Di Giovine G., Bollati M., Testa L., Bedogni F., Camporeale A., Pontone G., Andreini D., Monti L., Gasparini G. (2018). Cardiac magnetic resonance for ischaemia and viability detection. Guiding patient selection to revascularization in coronary chronic total occlusions: The CARISMA_CTO study design. Int. J. Cardiol..

[26] Raman S.V., Aneja A., Jarjour W.N. (2012). CMR in inflammatory vasculitis. J. Cardiovasc. Magn. Reson. Off. J. Soc. Cardiovasc. Magn. Reson..

[27] Mavrogeni S., Papadopoulos G., Douskou M., Kaklis S., Seimenis I., Baras P., Nikolaidou P., Bakoula C., Karanasios E., Manginas A. (2004). Magnetic resonance angiography is equivalent to X-ray coronary angiography for the evaluation of coronary arteries in Kawasaki disease. J. Am. Coll. Cardiol..

[28] Buechel E.R., Balmer C., Bauersfeld U., Kellenberger C.J., Schwitter J. (2009). Feasibility of perfusion cardiovascular magnetic resonance in paediatric patients. J. Cardiovasc. Magn. Reson. Off. J. Soc. Cardiovasc. Magn. Reson..

[29] Simonneau G., Montani D., Celermajer D.S., Denton C.P., Gatzoulis M.A., Krowka M., Williams P.G., Souza R. (2019). Haemodynamic definitions and updated clinical classification of pulmonary hypertension. Eur. Respir. J..

[30] Lechartier B., Chaouat A., Aubert J.D., Schwitter J. (2022). Magnetic resonance imaging in pulmonary hypertension: An overview of current applications and future perspectives. Swiss Med. Wkly..

[31] Alabed S., Garg P., Johns C.S., Alandejani F., Shahin Y., Dwivedi K., Zafar H., Wild J.M., Kiely D.G., Swift A.J. (2020). Cardiac Magnetic Resonance in Pulmonary Hypertension-an Update. Curr. Cardiovasc. Imaging Rep..

[32] Johns C.S., Kiely D.G., Rajaram S., Hill C., Thomas S., Karunasaagarar K., Garg P., Hamilton N., Solanki R., Capener D.A. (2019). Diagnosis of Pulmonary Hypertension with Cardiac MRI: Derivation and Validation of Regression Models. Radiology.

[33] Cerne J.W., Pathrose A., Gordon D.Z., Sarnari R., Veer M., Blaisdell J., Allen B.D., Avery R., Markl M., Ragin A. (2022). Evaluation of Pulmonary Hypertension Using 4D Flow MRI. J. Magn. Reson. Imaging.

[34] Mathew R.C., Löffler A.I., Salerno M. (2018). Role of Cardiac Magnetic Resonance Imaging in Valvular Heart Disease: Diagnosis, Assessment, and Management. Curr. Cardiol. Rep..

[35] Lopez-Mattei J.C., Shah D.J. (2013). The role of cardiac magnetic resonance in valvular heart disease. Methodist DeBakey Cardiovasc. J..

[36] Myerson S.G. (2021). CMR in Evaluating Valvular Heart Disease: Diagnosis, Severity, and Outcomes. JACC. Cardiovasc. Imaging.

[37] Anderson L.J., Holden S., Davis B., Prescott E., Charrier C.C., Bunce N.H., Firmin D.N., Wonke B., Porter J., Walker J.M. (2001). Cardiovascular T2-star (T2*) magnetic resonance for the early diagnosis of myocardial iron overload. Eur. Heart J..

[38] Torlasco C., Cassinerio E., Roghi A., Faini A., Capecchi M., Abdel-Gadir A., Giannattasio C., Parati G., Moon J.C., Cappellini M.D. (2018). Role of T1 mapping as a complementary tool to T2* for non-invasive cardiac iron overload assessment. PLoS ONE.

[39] Little J.T., Bookwalter C.A. (2020). Magnetic Resonance Safety: Pregnancy and Lactation. Magn. Reson. Imaging Clin. N. Am..

[40] Moschetti K., Kwong R.Y., Petersen S.E., Lombardi M., Garot J., Atar D., Rademakers F.E., Sierra-Galan L.M., Mavrogeni S., Li K. (2022). Cost-Minimization Analysis for Cardiac Revascularization in 12 Health Care Systems Based on the EuroCMR/SPINS Registries. JACC. Cardiovasc. Imaging.

[41] Muiño-Mosquera L., De Wilde H., Devos D., Babin D., Jordaens L., Demolder A., De Groote K., De Wolf D., De Backer J. (2020). Myocardial disease and ventricular arrhythmia in Marfan syndrome: A prospective study. Orphanet J. Rare Dis..

[42] Winther S., Williams L.K., Keir M., Connelly K.A., Bradley T.J., Rakowski H., Crean A.M. (2019). Cardiovascular Magnetic Resonance Provides Evidence of Abnormal Myocardial Strain and Primary Cardiomyopathy in Marfan syndrome. J. Comput. Assist. Tomogr..

[43] Merlocco A., Lacro R.V., Gauvreau K., Rabideau N., Singh M.N., Prakash A. (2017). Longitudinal Changes in Segmental Aortic Stiffness Determined by Cardiac Magnetic Resonance in Children and Young Adults With Connective Tissue Disorders (the Marfan, Loeys-Dietz, and Ehlers-Danlos Syndromes, and Nonspecific Connective Tissue Disorders). Am. J. Cardiol..

[44] Veldhoen S., Behzadi C., Lenz A., Henes F.O., Rybczynski M., von Kodolitsch Y., Bley T.A., Adam G., Bannas P. (2017). Non-contrast MR angiography at 1.5 Tesla for aortic monitoring in Marfan patients after aortic root surgery. J. Cardiovasc. Magn. Reson. Off. J. Soc. Cardiovasc. Magn. Reson..

[45] Somerville S., Rosolowsky E., Suntratonpipat S., Girgis R., Goot B.H., Tham E.B. (2016). Cardiac Magnetic Resonance Imaging in Pediatric Turner Syndrome. J. Pediatr..

[46] Mavrogeni S.I., Dimitroulas T., Kitas G.D. (2019). Cardiovascular magnetic resonance in the diagnosis and management of cardiac and vascular involvement in the systemic vasculitides. Curr. Opin. Rheumatol..

[47] Mavrogeni S., Dimitroulas T., Chatziioannou S.N., Kitas G. (2013). The role of multimodality imaging in the evaluation of Takayasu arteritis. Semin. Arthritis Rheum..

[48] Mavrogeni S., Papadopoulos G., Hussain T., Chiribiri A., Botnar R., Greil G.F. (2013). The emerging role of cardiovascular magnetic resonance in the evaluation of Kawasaki disease. Int. J. Cardiovasc. Imaging.

[49] Mavrogeni S., Karabela G., Gialafos E., Stavropoulos E., Spiliotis G., Katsifis G., Kolovou G. (2013). Cardiac involvement in ANCA (+) and ANCA (-) Churg-Strauss syndrome evaluated by cardiovascular magnetic resonance. Inflamm. Allergy Drug Targets.

[50] Bhalgat P., Naik A., Salvi P., Bhadane N., Shah K., Paunipagar B., Joshi S. (2018). Cardiac magnetic resonance imaging, myocardial scar and coronary flow pattern in anomalous origin of left coronary artery from the pulmonary artery. Indian Heart J..

[51] Nollen G.J., Kodde J., Beek A.M., Res J.C., van Rossum A.C. (2013). Quadricuspid pulmonary valve and left pulmonary artery aneurysm in an asymptomatic patient assessed by cardiovascular MRI. Neth. Heart J..

[52] Kato A., Drolet C., Yoo S.J., Redington A.N., Grosse-Wortmann L. (2016). Vicious circle between progressive right ventricular dilatation and pulmonary regurgitation in patients after tetralogy of Fallot repair? Right heart enlargement promotes flow reversal in the left pulmonary artery. J. Cardiovasc. Magn. Reson. Off. J. Soc. Cardiovasc. Magn. Reson..

[53] Latus H., Gummel K., Rupp S., Mueller M., Jux C., Kerst G., Akintuerk H., Bauer J., Schranz D., Apitz C. (2014). Cardiovascular magnetic resonance assessment of ventricular function and myocardial scarring before and early after repair of anomalous left coronary artery from the pulmonary artery. J. Cardiovasc. Magn. Reson. Off. J. Soc. Cardiovasc. Magn. Reson..

[54] Reiter U., Reiter G., Fuchsjäger M. (2016). MR phase-contrast imaging in pulmonary hypertension. Br. J. Radiol..

[55] Alabed S., Shahin Y., Garg P., Alandejani F., Johns C.S., Lewis R.A., Condliffe R., Wild J.M., Kiely D.G., Swift A.J. (2021). Cardiac-MRI Predicts Clinical Worsening and Mortality in Pulmonary Arterial Hypertension: A Systematic Review and Meta-Analysis. JACC Cardiovasc. Imaging.

[56] Ramos J.G., Fyrdahl A., Wieslander B., Reiter G., Reiter U., Jin N., Maret E., Eriksson M., Caidahl K., Sörensson P. (2020). Cardiovascular magnetic resonance 4D flow analysis has a higher diagnostic yield than Doppler echocardiography for detecting increased pulmonary artery pressure. BMC Med. Imaging.

[57] Patel A.R., Kramer C.M. (2017). Role of Cardiac Magnetic Resonance in the Diagnosis and Prognosis of Nonischemic Cardiomyopathy. JACC Cardiovasc. Imaging.

[58] Nanjo S., Yoshikawa K., Harada M., Inoue Y., Namiki A., Nakano H., Yamazaki J. (2009). Correlation between left ventricular diastolic function and ejection fraction in dilated cardiomyopathy using magnetic resonance imaging with late gadolinium enhancement. Circ. J. Off. J. Jpn. Circ. Soc..

[59] Becker M.A.J., Cornel J.H., van de Ven P.M., van Rossum A.C., Allaart C.P., Germans T. (2018). The Prognostic Value of Late Gadolinium-Enhanced Cardiac Magnetic Resonance Imaging in Nonischemic Dilated Cardiomyopathy: A Review and Meta-Analysis. JACC Cardiovasc. Imaging.

[60] Captur G., Arbustini E., Bonne G., Syrris P., Mills K., Wahbi K., Mohiddin S.A., McKenna W.J., Pettit S., Ho C.Y. (2018). Lamin and the heart. Heart.

[61] Holmström M., Kivistö S., Heliö T., Jurkko R., Kaartinen M., Antila M., Reissell E., Kuusisto J., Kärkkäinen S., Peuhkurinen K. (2011). Late gadolinium enhanced cardiovascular magnetic resonance of lamin A/C gene mutation related dilated cardiomyopathy. J. Cardiovasc. Magn. Reson. Off. J. Soc. Cardiovasc. Magn. Reson..

[62] Mavrogeni S., Markousis-Mavrogenis G., Papavasiliou A., Kolovou G. (2015). Cardiac involvement in Duchenne and Becker muscular dystrophy. World J. Cardiol..

[63] Brenes J.C., Doltra A., Prat S. (2018). Cardiac magnetic resonance imaging in the evaluation of patients with hypertrophic cardiomyopathy. Glob. Cardiol. Sci. Pract..

[64] Kamal M.U., Riaz I.B., Janardhanan R. (2016). Cardiovascular magnetic resonance imaging in hypertrophic cardiomyopathy: Current state of the art. Cardiol. J..

[65] Arcari L., Hinojar R., Engel J., Freiwald T., Platschek S., Zainal H., Zhou H., Vasquez M., Keller T., Rolf A. (2020). Native T1 and T2 provide distinctive signatures in hypertrophic cardiac conditions—Comparison of uremic, hypertensive and hypertrophic cardiomyopathy. Int. J. Cardiol..

[66] Pica S., Sado D.M., Maestrini V., Fontana M., White S.K., Treibel T., Captur G., Anderson S., Piechnik S.K., Robson M.D. (2014). Reproducibility of native myocardial T1 mapping in the assessment of Fabry disease and its role in early detection of cardiac involvement by cardiovascular magnetic resonance. J. Cardiovasc. Magn. Reson. Off. J. Soc. Cardiovasc. Magn. Reson..

[67] Hinojar R., Varma N., Child N., Goodman B., Jabbour A., Yu C.Y., Gebker R., Doltra A., Kelle S., Khan S. (2015). T1 Mapping in Discrimination of Hypertrophic Phenotypes: Hypertensive Heart Disease and Hypertrophic Cardiomyopathy: Findings From the International T1 Multicenter Cardiovascular Magnetic Resonance Study. Circulation. Cardiovasc. Imaging.

[68] Galea N., Polizzi G., Gatti M., Cundari G., Figuera M., Faletti R. (2020). Cardiovascular magnetic resonance (CMR) in restrictive cardiomyopathies. La Radiol. Med..

[69] Kirk P., Roughton M., Porter J.B., Walker J.M., Tanner M.A., Patel J., Wu D., Taylor J., Westwood M.A., Anderson L.J. (2009). Cardiac T2* magnetic resonance for prediction of cardiac complications in thalassemia major. Circulation.

[70] León D., Martín M., Corros C., Santamarta E., Costilla S., Lambert J.L. (2012). Usefulness of cardiac MRI in the early diagnosis of endomyocardial fibrosis. Rev. Port. De Cardiol..

[71] Pizarro C., Goebel A., Dabir D., Hammerstingl C., Pabst S., Grohé C., Fimmers R., Stoffel-Wagner B., Nickenig G., Schild H. (2016). Cardiovascular magnetic resonance-guided diagnosis of cardiac affection in a Caucasian sarcoidosis population. Sarcoidosis Vasc. Diffus. Lung Dis. Off. J. WASOG.

[72] Vignaux O., Dhote R., Duboc D., Blanche P., Dusser D., Weber S., Legmann P. (2002). Clinical significance of myocardial magnetic resonance abnormalities in patients with sarcoidosis: A 1-year follow-up study. Chest.

[73] Greulich S., Deluigi C.C., Gloekler S., Wahl A., Zürn C., Kramer U., Nothnagel D., Bültel H., Schumm J., Grün S. (2013). CMR imaging predicts death and other adverse events in suspected cardiac sarcoidosis. JACC Cardiovasc. Imaging.

[74] Ise T., Hasegawa T., Morita Y., Yamada N., Funada A., Takahama H., Amaki M., Kanzaki H., Okamura H., Kamakura S. (2014). Extensive late gadolinium enhancement on cardiovascular magnetic resonance predicts adverse outcomes and lack of improvement in LV function after steroid therapy in cardiac sarcoidosis. Heart.

[75] Zhuang X.F., Yang Y.M., Sun X.L., Liao Z.K., Huang J. (2017). Late onset radiation-induced constrictive pericarditis and cardiomyopathy after radiotherapy: A case report. Medicine.

[76] Mukai-Yatagai N., Haruki N., Kinugasa Y., Ohta Y., Ishibashi-Ueda H., Akasaka T., Kato M., Ogawa T., Yamamoto K. (2018). Assessment of myocardial fibrosis using T1-mapping and extracellular volume measurement on cardiac magnetic resonance imaging for the diagnosis of radiation-induced cardiomyopathy. J. Cardiol. Cases.

[77] Rathi V.K., Doyle M., Yamrozik J., Williams R.B., Caruppannan K., Truman C., Vido D., Biederman R.W. (2008). Routine evaluation of left ventricular diastolic function by cardiovascular magnetic resonance: A practical approach. J. Cardiovasc. Magn. Reson. Off. J. Soc. Cardiovasc. Magn. Reson..

[78] Vogelsberg H., Mahrholdt H., Deluigi C.C., Yilmaz A., Kispert E.M., Greulich S., Klingel K., Kandolf R., Sechtem U. (2008). Cardiovascular magnetic resonance in clinically suspected cardiac amyloidosis: Noninvasive imaging compared to endomyocardial biopsy. J. Am. Coll. Cardiol..

[79] Syed I.S., Glockner J.F., Feng D., Araoz P.A., Martinez M.W., Edwards W.D., Gertz M.A., Dispenzieri A., Oh J.K., Bellavia D. (2010). Role of cardiac magnetic resonance imaging in the detection of cardiac amyloidosis. JACC Cardiovasc. Imaging.

[80] Fontana M., Pica S., Reant P., Abdel-Gadir A., Treibel T.A., Banypersad S.M., Maestrini V., Barcella W., Rosmini S., Bulluck H. (2015). Prognostic Value of Late Gadolinium Enhancement Cardiovascular Magnetic Resonance in Cardiac Amyloidosis. Circulation.

[81] Mahalingam H., Chacko B.R., Irodi A., Joseph E., Vimala L.R., Thomson V.S. (2018). Myocardial nulling pattern in cardiac amyloidosis on time of inversion scout magnetic resonance imaging sequence—A new observation of temporal variability. Indian J. Radiol. Imaging.

[82] Binder C., Duca F., Binder T., Rettl R., Dachs T.M., Seirer B., Camuz Ligios L., Dusik F., Capelle C., Qin H. (2021). Prognostic implications of pericardial and pleural effusion in patients with cardiac amyloidosis. Clin. Res. Cardiol. Off. J. Ger. Card. Soc..

[83] Gunasekara M.Y., Mezincescu A.M., Dawson D.K. (2020). An Update on Cardiac Magnetic Resonance Imaging in Takotsubo Cardiomyopathy. Curr. Cardiovasc. Imaging Rep..

[84] Naruse Y., Sato A., Kasahara K., Makino K., Sano M., Takeuchi Y., Nagasaka S., Wakabayashi Y., Katoh H., Satoh H. (2011). The clinical impact of late gadolinium enhancement in Takotsubo cardiomyopathy: Serial analysis of cardiovascular magnetic resonance images. J. Cardiovasc. Magn. Reson. Off. J. Soc. Cardiovasc. Magn. Reson..

[85] Grothoff M., Pachowsky M., Hoffmann J., Posch M., Klaassen S., Lehmkuhl L., Gutberlet M. (2012). Value of cardiovascular MR in diagnosing left ventricular non-compaction cardiomyopathy and in discriminating between other cardiomyopathies. Eur. Radiol..

[86] Cesar S. (2018). Neuromuscular diseases with hypertrophic cardiomyopathy. Glob. Cardiol. Sci. Pract..

[87] Mavrogeni S., Papavasiliou A., Giannakopoulou K., Markousis-Mavrogenis G., Pons M.R., Karanasios E., Nikas I., Papadopoulos G., Kolovou G., Chrousos G.P. (2017). Oedema-fibrosis in Duchenne Muscular Dystrophy: Role of cardiovascular magnetic resonance imaging. Eur. J. Clin. Investig..

[88] Mavrogeni S., Markousis-Mavrogenis G., Markussis V., Kolovou G. (2015). The Emerging Role of Cardiovascular Magnetic Resonance Imaging in the Evaluation of Metabolic Cardiomyopathies. Horm. Metab. Res..

[89] Christidi A., Mavrogeni S.I. (2022). Rare Metabolic and Endocrine Diseases with Cardiovascular Involvement: Insights from Cardiovascular Magnetic Resonance—A Review. Horm. Metab. Res..

[90] Markousis-Mavrogenis G., Sfikakis P.P., Koutsogeorgopoulou L., Dimitroulas T., Katsifis G., Giannakopoulou A., Voulgari P., Kolovou G., Kitas G.D., Mavrogeni S.I. (2021). Cardiovascular Magnetic Resonance Reveals Cardiac Pathophysiology in Autoimmune Rheumatic Diseases. Mediterr. J. Rheumatol..

[91] Babu-Narayan S.V., Giannakoulas G., Valente A.M., Li W., Gatzoulis M.A. (2016). Imaging of congenital heart disease in adults. Eur. Heart J..

[92] Burke A. (2008). Primary malignant cardiac tumors. Semin. Diagn. Pathol..

[93] Elbardissi A.W., Dearani J.A., Daly R.C., Mullany C.J., Orszulak T.A., Puga F.J., Schaff H.V. (2008). Survival after resection of primary cardiac tumors: A 48-year experience. Circulation.

[94] Bruce C.J. (2011). Cardiac tumours: Diagnosis and management. Heart.

[95] Pazos-López P., Pozo E., Siqueira M.E., García-Lunar I., Cham M., Jacobi A., Macaluso F., Fuster V., Narula J., Sanz J. (2014). Value of CMR for the differential diagnosis of cardiac masses. JACC Cardiovasc. Imaging.

[96] Ferreira V.M., Holloway C.J., Piechnik S.K., Karamitsos T.D., Neubauer S. (2013). Is it really fat? Ask a T1-map. Eur. Heart J. Cardiovasc. Imaging.

[97] Saunderson C.E.D., Plein S., Manisty C.H. (2021). Role of cardiovascular magnetic resonance imaging in cardio-oncology. Eur. Heart J. Cardiovasc. Imaging.

[98] Armstrong G.T., Plana J.C., Zhang N., Srivastava D., Green D.M., Ness K.K., Daniel Donovan F., Metzger M.L., Arevalo A., Durand J.B. (2012). Screening adult survivors of childhood cancer for cardiomyopathy: Comparison of echocardiography and cardiac magnetic resonance imaging. J. Clin. Oncol..

[99] Burrage M.K., Ferreira V.M. (2020). The use of cardiovascular magnetic resonance as an early non-invasive biomarker for cardiotoxicity in cardio-oncology. Cardiovasc. Diagn. Ther..

[100] Cau R., Solinas C., De Silva P., Lambertini M., Agostinetto E., Scartozzi M., Montisci R., Pontone G., Porcu M., Saba L. (2022). Role of cardiac MRI in the diagnosis of immune checkpoint inhibitor-associated myocarditis. Int. J. Cancer.

[101] Castrichini M., Eldemire R., Groves D.W., Taylor M.R., Miyamoto S., Mestroni L. (2021). Clinical and genetic features of arrhythmogenic cardiomyopathy: Diagnosis, management and the heart failure perspective. Prog. Pediatr. Cardiol..

[102] Marcus F.I., McKenna W.J., Sherrill D., Basso C., Bauce B., Bluemke D.A., Calkins H., Corrado D., Cox M.G., Daubert J.P. (2010). Diagnosis of arrhythmogenic right ventricular cardiomyopathy/dysplasia: Proposed modification of the Task Force Criteria. Eur. Heart J..

[103] He J., Xu J., Li G., Zhou D., Li S., Zhuang B., Chen X., Duan X., Li L., Fan X. (2020). Arrhythmogenic Left Ventricular Cardiomyopathy: A Clinical and CMR Study. Sci. Rep..

[104] Aquaro G.D., De Luca A., Cappelletto C., Raimondi F., Bianco F., Botto N., Lesizza P., Grigoratos C., Minati M., Dell’Omodarme M. (2020). Prognostic Value of Magnetic Resonance Phenotype in Patients With Arrhythmogenic Right Ventricular Cardiomyopathy. J. Am. Coll. Cardiol..

[105] Schaufelberger M. (2019). Cardiomyopathy and pregnancy. Heart.

[106] Nii M., Ishida M., Dohi K., Tanaka H., Kondo E., Ito M., Sakuma H., Ikeda T. (2018). Myocardial tissue characterization and strain analysis in healthy pregnant women using cardiovascular magnetic resonance native T1 mapping and feature tracking technique. J. Cardiovasc. Magn. Reson..

[107] Gopi A., Sebastian P., Ramakrishna C.D. (2016). A rare malady with a rarer complication. Indian Heart J..

[108] Soler R., Rodríguez E., Monserrat L., Alvarez N. (2002). MRI of subendocardial perfusion deficits in isolated left ventricular noncompaction. J. Comput. Assist. Tomogr..

